# Supplementary material for: Differentially Expressed Genes and Signaling Pathways Potentially Involved in Primary Resistance to Chemo-Immunotherapy in Advanced-Stage Gastric Cancer Patients
Source: Int J Mol Sci. 2022 Dec 20;24(1):1. doi: 10.3390/ijms24010001 (PMC9820415; doi:10.3390/ijms24010001)
Supplement: Supplementary file 1 [file ijms-24-00001-s001.zip › Supp Fig S1 copia.pdf]

Cutoff: p<0.001
53 differentially expressed genes

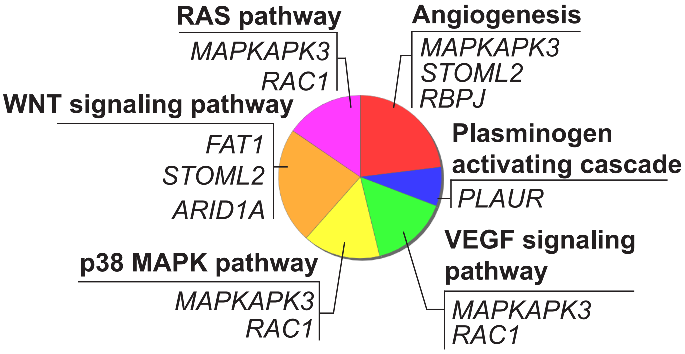

Supplementary Figure S1. Other enriched pathways in NR patients
