# Supplementary material for: Differentially Expressed Genes and Signaling Pathways Potentially Involved in Primary Resistance to Chemo-Immunotherapy in Advanced-Stage Gastric Cancer Patients
Source: Int J Mol Sci. 2022 Dec 20;24(1):1. doi: 10.3390/ijms24010001 (PMC9820415; doi:10.3390/ijms24010001)
Supplement: Supplementary file 1 [file ijms-24-00001-s001.zip › Suppl Table s1.pdf]

Supplementary Table S1. Basic characteristics and clinically relevant alterations in NR patients (n=4)

	**Patient 1**	**Patient 2**	**Patient 3**	**Patient 4**
Age (yr), gender	47, female	21, female	27, female	49, male
Diagnosis	Gastric carcinoma	Gastric carcinoma	Gastric carcinoma	Gastric carcinoma
Immuno response	Non-responder	Non-responder	Non-responder	Non-responder
Type of biopsy	Tissue	Tissue	Tissue	Tissue
Relevant alterations	***ARID1A***<br>(c.6472C>T, p.Arg2158Ter)<br>***PIK3CA***<br>(c.3140A>G, p.His1047Arg)<br>***ERBB2/HER2***<br>(c.929C>T, p.Ser310Phe)<br>***ESR1***(2)-***CCDC170***(8)<br>gene fusion	***TP53***<br>(c.159G>A, p.W53*)<br>***PIK3CA***<br>(c.1636C>A, p.Q546K)<br>***SMAD4***<br>(c.1054G>A, p.G352R)<br>***PBRM1***<br>(c.4132+1G>A)<br>***CDK6***<br>copy number gain	***TP53***<br>(c.692_700dupCCATCCACT, p.H233_Y234insSIH)<br>***PIK3R1***<br>(c.328C>T, p.Q110*)<br>***SMAD4***<br>(c.904+1_904+2insGCCTGTTCACAATGAGCTTGCATTCCAGCCTCCCATTTCCAATCA)<br>***TP53***<br>copy number loss	***ARID1A***<br>(c.1554C>G, p.Y518*)<br>***TP53***<br>(c.734G>T, p.G245V)<br>***TP53***<br>(c.578A>G, p.H193R)<br>***APC***<br>(c.3451G>T, p.E1151*)
